# Cartilage lesion size and number of stromal vascular fraction (SVF) cells strongly influenced the SVF implantation outcomes in patients with knee osteoarthritis

**DOI:** 10.1186/s40634-023-00592-1

**Published:** 2023-03-15

**Authors:** Yong Sang Kim, Sun Mi Oh, Dong Suk Suh, Dae Hyun Tak, Yoo Beom Kwon, Yong Gon Koh

**Affiliations:** Center for Stem Cell & Arthritis Research, Department of Orthopaedic Surgery, Yonsei Sarang Hospital, 10, Hyoryeong-Ro, Seocho-Gu, Seoul, 06698 Republic of Korea

**Keywords:** Stromal vascular fraction, Prognostic factors, Osteoarthritis, Knee

## Abstract

**Purpose:**

This study evaluated outcomes in patients with knee osteoarthritis following stromal vascular fraction implantation and assessed the associated prognostic factors.

**Methods:**

We retrospectively evaluated 43 patients who underwent follow-up magnetic resonance imaging 12 months after stromal vascular fraction implantation for knee osteoarthritis. Pain was assessed using the visual analogue scale and measured at baseline and 1-, 3-, 6-, and 12-month follow-up appointments. In addition, cartilage repair was evaluated based on the Magnetic Resonance Observation of Cartilage Repair Tissue scoring system using the magnetic resonance imaging from the 12-month follow-up. Finally, we evaluated the effects of various factors on outcomes following stromal vascular fraction implantation.

**Results:**

Compared to the baseline value, the mean visual analogue scale score significantly and progressively decreased until 12 months post-treatment (*P* < 0.05 for all, except n.s. between the 1 and 3-month follow-ups). The mean Magnetic Resonance Observation of Cartilage Repair Tissue score was 70.5 ± 11.1. Furthermore, the mean visual analogue scale and Magnetic Resonance Observation of Cartilage Repair Tissue scores significantly correlated 12 months postoperatively (*P* = 0.002). Additionally, the cartilage lesion size and the number of stromal vascular fraction cells significantly correlated with the 12-month visual analogue scale scores and the Magnetic Resonance Observation of Cartilage Repair Tissue score. Multivariate analyses determined that the cartilage lesion size and the number of stromal vascular fraction cells had a high prognostic significance for unsatisfactory outcomes.

**Conclusion:**

Stromal vascular fraction implantation improved pain and cartilage regeneration for patients with knee osteoarthritis. The cartilage lesion size and the number of stromal vascular fraction cells significantly influenced the postoperative outcomes. Thus, these findings may serve as a basis for preoperative surgical decisions.

**Level of evidence:**

IV.

## Introduction

Osteoarthritis (OA) is an increasingly prevalent, progressive, and painful chronic joint disorder accompanied by deteriorating joint function [16]. The knee is the principally affected peripheral joint, resulting in pain, stiffness, and progressive loss of function [10]. Knee OA is a painful and debilitating process that significantly affects the patient’s quality of life [3]. The poor intrinsic healing potential of damaged cartilage, which results in progressive degradation of articular cartilage and subsequent widespread degeneration of the joint, is a major clinical problem in knee OA treatment [13]. Hence, restoring the diseased articular cartilage in patients with knee OA is a challenging but important problem for researchers and clinicians [30].

Recently, cell-based therapies have emerged as potential treatment options for managing knee OA [26]. Mesenchymal stem cells (MSCs) from various sources have been extensively evaluated for their ability to restore compromised articular cartilage and slow knee OA progression [44]. The pathogenesis of OA is based on degeneration and inflammation. Thus, the therapeutic properties of MSCs, including paracrine [6, 20], anti-inflammatory [39], and immunomodulatory effects [40], could help restore the intra-articular environment [31]. However, MSCs require culturing, including a few weeks between cell isolation and application, and is also expensive.

Alternatively, adipose-derived stromal vascular fraction (SVF) has received more attention as a stem cell source for managing knee OA at any stage, as lipoaspirates are easy to obtain using a minimally invasive procedure with a low complication rate and minimal donor-site morbidity [17, 41]. Adipose-derived SVF cells are a heterogeneous cell population containing regenerative cells (such as adipose-derived MSCs), macrophages, pericytes, fibroblasts, blood cells, vessel-forming cells (including endothelial and smooth muscle cells), and their progenitors [19]. This heterogeneous cell population includes cells with stem cell elements and is thought to have a synergistic effect with adipose-derived MSCs [37]. Furthermore, adipose-derived SVF and MSCs both result in comparable clinical improvement in patients with knee OA [41].

Several studies have used adipose-derived SVF for knee OA treatment [5, 11, 41, 42]. However, to date, none have assessed factors that influence the outcomes of SVF-based treatment for knee OA. Identifying factors associated with favourable and unfavourable outcomes would provide patients with realistic expectations of outcomes after SVF-based treatment [34]. Accordingly, this study investigated the pain relief and cartilage repair status after arthroscopic SVF implantation in patients with knee OA to identify prognostic factors associated with outcomes. We hypothesised that some factors increase the risk of an unsatisfactory outcome.

## Materials and methods

### Patient enrolment

We retrospectively reviewed the medical records of 62 consecutive patients with a 12-month follow-up period who underwent arthroscopic SVF implantation for knee OA between September 2019 and April 2021. Our institutional review board reviewed and approved this study. Furthermore, the study was supported by the ‘Conditional Approval System of Health Technology’ grant, funded by the Ministry of Health and Welfare. The study is the result of analysing the parts of participants among the all subjects who were participated in ‘Conditional Approval System of Health Technology’ grant. All participants provided informed consent prior to enrolment.

Medical records and plain radiographs were assessed, and patients with symptomatic knee pain unresponsive to nonoperative treatment were included. The exclusion criteria were previous surgical treatment, knee instability, knee varus or valgus malalignment, and other pathological diseases, including rheumatoid arthritis, haemophilia, and active knee infections. We suggested that all patients undergo follow-up magnetic resonance imaging (MRI), explaining its purpose (to evaluate the cartilage lesion and other pathologic conditions) before surgery. Of the 62 qualified patients, 14 dropped out and 5 were lost during the follow-up. Therefore, 43 patients were enrolled, including 14 men and 29 women with a mean age of 63.4 (range, 53–74) years. The average preoperative body mass index (BMI) was 26.0 (range, 19.5–32.5) kg/m^2^, and the mean cartilage lesion size was 5.6 (range, 3.2–7.9) cm^2^ (Table 1).

**Table 1:** Baseline characteristics

Age, y	63.4 ± 4.1 (53–74)
Sex, male/female, n	14/29
Side of involvement, right/left, n	21/22
Body mass index, kg/m^2^	26.0 ± 2.8 (19.5–32.5)
Lesion size, cm^2^	5.6 ± 1.3 (3.2–7.9)

Data are presented as means ± standard deviation (range) unless otherwise indicated

### SVF preparation and surgical procedures

One day prior to SVF implantation, samples of adipose tissue were collected from the gluteal regions of the study participants. The collected adipose tissue was suspended in phosphate-buffered saline solution and transported to the laboratory in a sterile box. Prior to implantation, mature adipocytes and connective tissues were separated from the SVF by centrifugation [46], and bacteriologic tests were performed to ensure that the samples were not contaminated; cell viability was assessed using the methylene blue dye exclusion test. A certain amount of adipose tissue was used for cell analyses. After isolating and characterising the adipose-derived cells as described previously [22, 23, 27], we confirmed that they contained MSCs. The isolation and characterisation procedures determined that adipose-derived stem cells made up 9.5% (range, 8.6–11.2%) of the SVF. Consequently, an average of 7.4 × 10^7^ cells (range, 6.7 × 10^7^–8.5 × 10^7^ cells) in the SVF, which contained an average of 7.0 × 10^6^ stem cells (range, 6.4 × 10^6^–8.1 × 10^6^ cells), were used for SVF implantation.

Before SVF implantation, arthroscopic debridement of the damaged or undermined cartilage was performed to smooth the cartilage lesion surface and firm up the edges facing the surrounding cartilage. Before SVF implantation, subchondral drilling was performed to increase the adhesion rate of the applied SVF mixed with fibrin glue. The prepared SVF was loaded into the fibrin glue product from the commercially available Greenplast kit (Greencross, Seoul, Korea), which was used as a scaffold for SVF implantation. After the arthroscopic fluid was extracted, the prepared SVF loaded into the fibrin glue was implanted into the cartilage lesion site under arthroscopic guidance. Then, the applied SVF mixed with fibrin glue was manipulated using the probe to cover the surface of the cartilage lesion evenly. After performing the arthroscopic procedure, the knee was immobilised for two weeks with a knee brace. After the sutures were removed, the patients began range of motion exercises, including active and passive knee joint exercises. Partial weight-bearing activities were initiated two weeks after arthroscopy, and full weight-bearing activities were permitted four weeks postoperatively. Sports and high-impact activities were allowed after three months, and the full return to regular sports or recreational activities was permitted based on the patients’ individual recovery.

### Outcome assessment

All patients were clinically evaluated preoperatively and 1, 3, 6, and 12 months postoperatively at follow-up visits. The visual analogue scale (VAS; range, 0–100) was used for pain assessment and was measured over the follow-up period. Adverse events were recorded for safety evaluation. A follow-up MRI was performed 12 months postoperatively using a 3.0 T MRI scanner. To avoid potential bias, an independent observer, who was a radiologist not involved in patient care and blinded to the study’s purpose, evaluated the MRI scans. Repair tissue evaluations were performed using the follow-up MRI and the Magnetic Resonance Observation of Cartilage Repair Tissue (MOCART) scoring system, according to Marlovits et al. [28] (Table 2).

**Table 2:** The MOCART scores based on the 12-month follow-up MRI examination

Variables	Score	n	Mean ± SD	95% CI
1. Degree of defect repair and filling of the defect			17.9 ± 3.1	16.94 – 18.87
Complete	20	27		
Hypertrophy	15	15		
Incomplete				
> 50% of the adjacent cartilage	10	0		
< 50% of the adjacent cartilage	5	1		
Subchondral bone exposed	0	0		
2. Integration to border zone			7.7 ± 2.7	6.83 – 8.52
Complete	15	1		
Incomplete				
Demarcating border visible	10	21		
Defect visible				
< 50% of the length of the repair tissue	5	21		
> 50% of the length of the repair tissue	0	0		
3. Surface of the repair tissue			2.6 ± 3.2	1.59 – 3.53
Surface intact	10	3		
Surface damaged				
< 50% of repair tissue depth or total degeneration	5	26		
> 50% of repair tissue depth or total degeneration	0	24		
4. Structure of the repair tissue			4.9 ± 0.7	4.65 – 5.12
Homogenous	5	42		
Inhomogenous or cleft formation	0	1		
5. Signal intensity of the repair tissue			21.6 ± 7.5	19.31 – 23.95
Normal (identical to adjacent cartilage)	30	19		
Nearly normal (slight areas of signal alteration)	15	24		
Abnormal (large areas of signal alteration)	0	0		
6. Subchondral lamina			4.9 ± 0.8	4.65 – 5.12
Intact	5	42		
Not intact	0	1		
7. Subchondral bone			4.8 ± 1.1	4.44 – 5.10
Intact	5	41		
Not intact	0	2		
8. Adhesions			3.5 ± 2.3	2.77 – 4.20
No	5	30		
Yes	0	13		
9. Effusion			1.9 ± 2.4	1.11 – 2.61
No	5	26		
Yes	0	27		
Total	100		70.5 ± 11.1	67.06 – 73.87

Data are presented as means ± standard deviation (SD) unless otherwise indicated

*MOCART* Magnetic Resonance Observation of Cartilage Repair Tissue, *MRI* Magnetic resonance imaging, *SD* Standard deviation, *CI* Confidence interval

### Statistical analyses

The principal dependent variables were the VAS scores during the follow-up visits and the MOCART score. Descriptive statistics were calculated as means ± standard deviations unless otherwise indicated. The Wilcoxon signed-rank test was used to evaluate differences between the preoperative and final follow-up values. We divided the patients into subgroups to assess various factors that may influence the outcomes: age (< 60, 60–65, 65–70, and ≥ 70 years), sex (male and female), involved side (right and left), BMI (< 20, 20.0–24.9, 25–29.9, and ≥ 30.0 kg/m^2^), lesion location (medial femoral condyle, lateral femoral condyle, and trochlea), lesion size (< 3.5, 3.5–5.5, 5.5–7.5, and ≥ 7.5 cm^2^), and the number of SVF cells (< 7.0 × 10^7^, 7.0 × 10^7^–8.0 × 10^7^, and ≥ 8.0 × 10^7^). Differences between the groups were analysed using the Mann–Whitney U test or the Kruskal–Wallis test for multiple comparisons. The Spearman’s rank-order correlation test was used to evaluate potential bivariate associations between different factors to identify significant correlations. Multivariate logistic regression analyses were used to determine factors independently associated with unsatisfactory outcomes. We defined an unsatisfactory outcome as a VAS score of < 35.9 based on the mean VAS score at the 12-month follow-up (i.e., 35.9) and a MOCART score of < 70.5 based on the mean MOCART score (i.e., 70.5). We calculated odds ratios and 95% confidence intervals (CIs) relative to a chosen reference group for the logistic regression models. Statistical analyses were performed using SPSS, Version 13.0 (IBM Corp., Armonk, NY, USA), and a *P*-value of < 0.05 was considered statistically significant.

## Results

### Pain scores and MRI outcomes

**Fig. 1:**
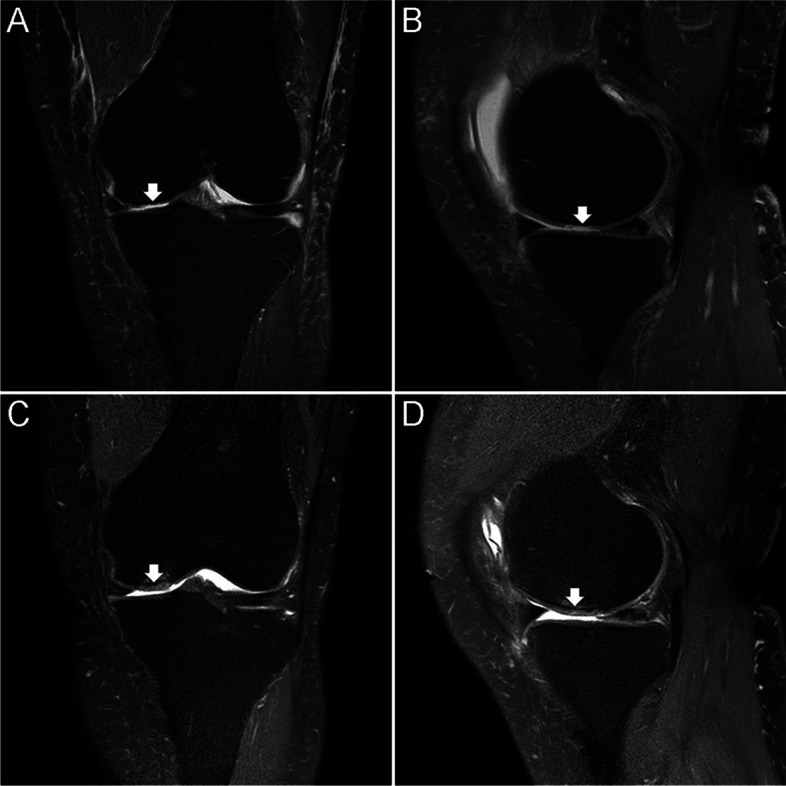
Preoperative (**A** and **B**) and follow-up (**C** and **D**) coronal and sagittal proton density fat-saturated images of the right knee of a 64-year-old female patient. **A** and **B** Cartilage loss is visible in the medial femoral condyle (arrows). **C** and **D** Complete filling of the defect along with complete integration with the adjacent native cartilage (arrows; MOCART score, 75 points)

The mean VAS scores at baseline and 1, 3, 6, and 12 months postoperatively were 79.1 ± 6.9, 43.5 ± 8.6, 43.3 ± 9.3, 40.2 ± 8.8, and 35.9 ± 7.1, respectively. The mean VAS score after 1 month was significantly lower than the mean baseline score (*P* < 0.001). The mean VAS score did not differ between months 1 and 3 (n.s.), but otherwise, they significantly and progressively decreased during the follow-up period until 12 months post-treatment (all *P* < 0.001). The mean MOCART score after 12 months was 70.5 ± 11.1 (Table 2) (Fig. 1). The mean VAS and MOCART scores did not correlate until 6 months after surgery, but they significantly correlated 12 months after surgery (*P* = 0.002; Table 3).

**Table 3:** Pain score and MRI outcome correlations

	MOCART	
	*S* rho	*P* value
VAS		
Baseline	− 0.148	n.s
1 month	− 0.203	n.s
3 months	− 0.228	n.s
6 months	− 0.201	n.s
12 months	− 0.463	0.002

Data are calculated using the Spearman’s rank-order test

*MRI* Magnetic resonance imaging, *MOCART* Magnetic resonance observation of cartilage repair tissue, *VAS* Visual analogue scale

### Outcome associations

Tables 4, 5, 6 and 7 present the mean VAS and MOCART scores based on various factors, including age, sex, the involved side, BMI, and lesion location. The mean VAS and MOCART scores did not differ among the age, sex, involved side, BMI, lesion location groups (all n.s.).

**Table 4:** The pain and MOCART scores stratified by age

	Age, y				
	< 60 (*n* = 4)	60–65 (*n* = 24)	65–70 (*n* = 12)	≥ 70 (*n* = 3)	*P* value^*^
VAS					
Baseline	74.2 ± 5.1	80.1 ± 7.1	78.8 ± 6.4	78.8 ± 10.2	n.s
1 month	37.8 ± 11.0	45.2 ± 8.8	43.1 ± 7.8	39.3 ± 2.5	n.s
3 months	37.5 ± 13.0	45.8 ± 9.5	42.0 ± 7.1	37.0 ± 5.3	n.s
6 months	34.0 ± 11.1	41.8 ± 8.6	–	39.0 ± 7.2	n.s
12 months	34.4 ± 11.0	35.7 ± 4.8	36.1 ± 7.9	38.7 ± 15.5	n.s
MOCART	73.8 ± 10.3	73.3 ± 10.5	65.0 ± 9.5	65.0 ± 17.3	n.s

Data are presented as means ± standard deviation

*MOCART* Magnetic resonance observation of cartilage repair tissue, *VAS* Visual analogue scale

^*^Kruskal–Wallis test

**Table 5:** Pain and MOCART scores stratified by sex and the involved side

	Sex			Involved side		
	Male (*n* = 14)	Female (*n* = 29)	*P* value^*^	Right (*n* = 21)	Left (*n* = 22)	*P* value^*^
VAS						
Baseline	77.5 ± 6.6	79.8 ± 7.1	n.s	79.5 ± 6.3	78.7 ± 7.6	n.s
1 month	42.9 ± 9.9	43.8 ± 8.0	n.s	42.5 ± 8.2	44.5 ± 9.0	n.s
3 months	43.4 ± 10.0	43.3 ± 9.1	n.s	41.1 ± 8.7	45.5 ± 9.5	n.s
6 months	40.2 ± 11.7	40.2 ± 7.2	n.s	38.2 ± 7.6	42.2 ± 9.5	n.s
12 months	34.8 ± 7.7	36.4 ± 6.8	n.s	34.7 ± 6.5	37.0 ± 7.5	n.s
MOCART	72.5 ± 10.1	69.5 ± 11.5	n.s	69.8 ± 12.0	71.1 ± 10.3	n.s

Data are presented as means ± standard deviation

*MOCART* Magnetic resonance observation of cartilage repair tissue, *VAS* Visual analogue scale

^*^Mann–Whitney *U* test

**Table 6:** Pain and MOCART scores stratified by body mass index

	Body mass index, kg/m^2^				
	< 20.0 (*n* = 2)	20.0–24.9 (*n* = 15)	25.0–29.9 (*n* = 24)	≥ 30.0 (*n* = 2)	*P* value^*^
VAS					
Baseline	77.5 ± 0.7	79.9 ± 7.4	78.9 ± 7.0	77.0 ± 8.5	n.s
1 month	40.0 ± 5.7	44.7 ± 5.4	44.0 ± 10.1	32.5 ± 4.9	n.s
3 months	37.5 ± 7.8	44.0 ± 6.5	43.3 ± 10.9	45.0 ± 11.3	n.s
6 months	37.0 ± 8.5	39.9 ± 5.7	40.4 ± 10.4	43.5 ± 12.0	n.s
12 months	25.8 ± 13.0	36.4 ± 5.2	36.1 ± 7.5	40.0 ± 1.4	n.s
MOCART	67.5 ± 3.5	70.3 ± 12.9	71.0 ± 10.9	67.5 ± 3.5	n.s

Data are presented as means ± standard deviation

*MOCART* Magnetic resonance observation of cartilage repair tissue, *VAS* Visual analogue scale

^*^Kruskal–Wallis test

**Table 7:** Pain and MOCART scores stratified by lesion location

	Lesion location			
	Medial femoral condyle (*n* = 41)	Lateral femoral condyle (*n* = 16)	Trochlea (*n* = 6)	*P* value^*^
VAS				
Baseline	78.5 ± 7.1	77.1 ± 6.0	81.0 ± 6.7	n.s
1 month	46.7 ± 7.2	45.5 ± 7.7	50.4 ± 6.8	n.s
3 months	46.2 ± 8.1	44.7 ± 10.0	48.3 ± 8.5	n.s
6 months	41.5 ± 7.6	38.0 ± 10.9	44.8 ± 11.4	n.s
12 months	37.1 ± 7.2	33.5 ± 6.9	34.7 ± 8.0	n.s
MOCART	70.6 ± 10.7	75.6 ± 10.5	75.8 ± 12.4	n.s

Data are presented as means ± standard deviation

*MOCART* Magnetic resonance observation of cartilage repair tissue, *VAS* Visual analogue scale

^*^Kruskal–Wallis test

**Fig. 2:**
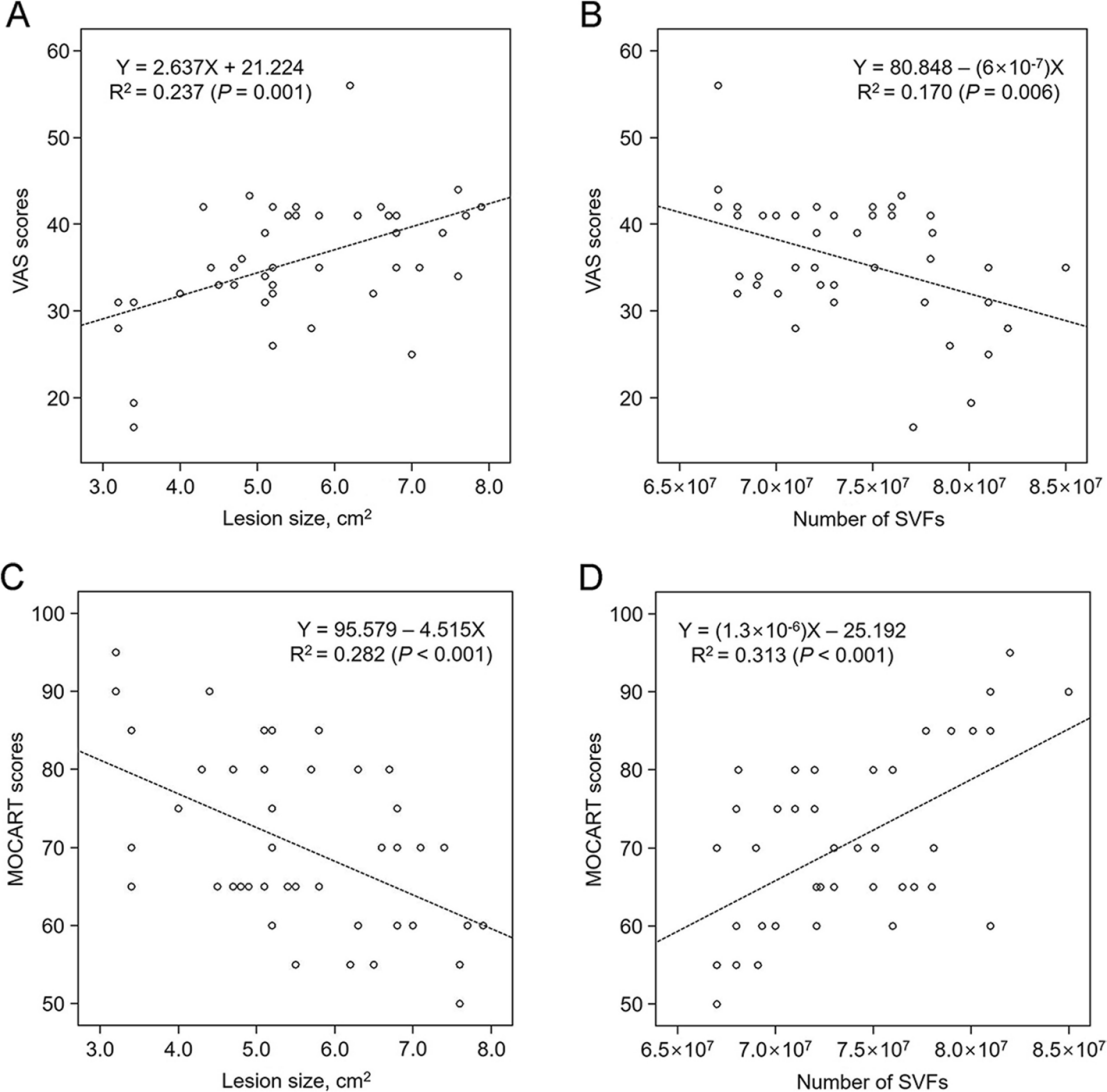
Correlations between the **A**, **B** 12-month visual analogue scale (VAS) and **C**, **D** magnetic resonance observation of cartilage repair tissue (MOCART) scores and the **A**, **C** lesion size and **B**, **D** the number of stromal vascular fraction (SVF) cells

The mean cartilage lesion size was 5.6 ± 1.3 (range, 3.2–7.9) cm^2^, and Table 8 presents the mean VAS and MOCART scores based on the lesion size. The mean VAS scores at 12 months significantly differed among the lesion size groups (*P* = 0.008), as did the mean MOCART scores (*P* = 0.007). Furthermore, the 12-month VAS score and the lesion size significantly correlated (Fig. 2A), as did the MOCART score and the lesion size (Fig. 2C).

**Table 8:** Pain and MOCART scores stratified by lesion size

	Lesion size, cm^2^				
	< 3.5 (*n* = 4)	3.5–5.5 (*n* = 18)	5.5–7.5 (*n* = 17)	≥ 7.5 (*n* = 4)	*P* value^*^
VAS					
Baseline	81.0 ± 4.8	78.1 ± 6.3	80.5 ± 7.3	75.6 ± 10.2	n.s
1 month	42.0 ± 4.7	43.3 ± 10.0	43.9 ± 8.4	44.3 ± 7.9	n.s
3 months	40.8 ± 5.1	42.1 ± 10.8	44.2 ± 9.2	47.8 ± 4.9	n.s
6 months	40.5 ± 6.4	38.2 ± 10.6	41.1 ± 8.0	40.8 ± 6.6	n.s
12 months	26.7 ± 6.8	34.5 ± 5.9	38.5 ± 6.8	40.3 ± 4.3	0.008
MOCART	80.0 ± 14.7	73.9 ± 9.2	67.9 ± 9.7	56.3 ± 4.8	0.007

Data are presented as means ± standard deviation

*MOCART* Magnetic resonance observation of cartilage repair tissue, *VAS* Visual analogue scale

^*^Kruskal–Wallis test

The mean number of SVF cells was 7.4 × 10^7^ ± 4.8 × 10^6^ (range, 6.7 × 10^7^–8.5 × 10^7^). Table 9 details the association between the number of SVF cells and patient characteristics, none of which correlated. Table 10 reports the mean VAS scores based on the number of SVF cells, and the 12-month mean VAS score significantly differed among the SVF groups (*P* = 0.022). We also identified a significant correlation between the 12-month VAS score and the number of SVF cells (Fig. 2B).

**Table 9:** Patient characteristics and SVF correlations

Patient Characteristics	Number of SVF cells	
	*S* rho	*P* value
Age	− 0.243	n.s
Sex	0.158	n.s
Side of involvement	− 0.008	n.s
Body mass index	− 0.012	n.s

Data are calculated using the Spearman’s rank-order test

*SVF* Stromal vascular fraction

**Table 10:** Pain scores stratified by the number of SVF cells

	Number of SVF cells			
	< 7.0 × 10^7^ (*n* = 12)	7.0 × 10^7^–8.0 × 10^7^ (*n* = 25)	≥ 8.0 × 10^7^ (*n* = 6)	*P* value^*^
VAS				
Baseline	79.1 ± 9.2	79.5 ± 6.0	77.4 ± 6.4	n.s
1 month	44.8 ± 8.1	42.8 ± 7.7	44.2 ± 13.3	n.s
3 months	45.8 ± 8.8	42.2 ± 8.1	43.4 ± 14.8	n.s
6 months	40.0 ± 7.6	40.5 ± 7.0	39.4 ± 16.8	n.s
12 months	39.3 ± 6.9	35.9 ± 6.3	28.9 ± 6.1	0.022

Data are presented as means ± standard deviation

*SVF* Stromal vascular fraction, *VAS* Visual analogue scale

^*^Kruskal–Wallis test

Table 11 presents the mean MOCART score based on the number of SVF cells. The mean MOCART score significantly differed among the SVF groups (*P* = 0.001). Furthermore, some variables comprising the MOCART score significantly differed among the SVF groups, such as the degree of defect repair and defect filling (*P* = 0.016), border zone integration (*P* = 0.018), the repair tissue surface (*P* = 0.043), and repair tissue signal intensity (*P* = 0.025). We also identified a significant correlation between the MOCART score and the number of SVF cells (Fig. 2D).

**Table 11:** The MOCART score stratified by the number of SVF cells

Variables	Number of SVF cells			
	< 7.0 × 10^7^ (*n* = 12)	7.0 × 10^7^–8.0 × 10^7^ (*n* = 25)	≥ 8.0 × 10^7^ (*n* = 6)	*P* value^*^
Degree of defect repair and filling of the defect	15.8 ± 4.2	19.0 ± 2.0	17.5 ± 2.7	0.016
Integration to border zone	7.1 ± 2.6	7.2 ± 2.5	10.8 ± 2.0	0.018
Surface of the repair tissue	1.7 ± 2.5	2.2 ± 2.9	5.8 ± 3.8	0.043
Structure of the repair tissue	5.0 ± 0.0	4.8 ± 1.0	5.0 ± 0.0	n.s
Signal intensity of the repair tissue	17.5 ± 5.8	22.2 ± 7.6	27.5 ± 6.1	0.025
Subchondral lamina	4.6 ± 1.4	5.0 ± 0.0	5.0 ± 0.0	n.s
Subchondral bone	5.0 ± 0.0	4.6 ± 1.4	5.0 ± 0.0	n.s
Adhesions	2.9 ± 2.6	3.6 ± 2.3	4.2 ± 2.0	n.s
Effusion	1.7 ± 2.5	1.6 ± 2.4	3.3 ± 2.6	n.s
Total	62.1 ± 9.4	71.2 ± 7.7	84.2 ± 12.4	0.001

Data are presented as means ± standard deviation

*MOCART* Magnetic resonance observation of cartilage repair tissue, *SVF* Stromal vascular fraction

^*^Kruskal–Wallis test

Multivariate logistic regression analyses were used to identify factors independently associated with unsatisfactory outcomes. Table 12 presents the final model, which controlled for age, sex, the involved side, BMI, lesion size, and the number of SVF cells. The lesion size and the number of SVF cells were independent predictors of an unsatisfactory outcome after SVF implantation (*P* = 0.038 and 0.021, respectively). Compared to patients with a lesion < 3.5 cm^2^, those with a 3.5 to 5.5 cm^2^ lesion were 2.67 times more likely to have an unsatisfactory outcome (95% CI, 0.23–31.07). Meanwhile, patients with a 5.5 to 7.5 cm^2^ lesion were 7.80 times more likely to have an unsatisfactory outcome (95% CI, 0.65–93.81), and those with a lesion ≥ 7.5 cm^2^ were 13.4 times more likely to have an unsatisfactory outcome (95% CI, 2.49–215.36).

**Table 12:** Associations between patient factors and an unsatisfactory outcome after SVF implantation

Factors	n (%)	Unsatisfactory outcome, odds ratio (95% CI)	*P* value
Age, y			n.s
< 60	4 (9.3)	1.50 (0.06–40.63)	
60–65	24 (55.8)	1.21 (0.09–145.66)	
65–70	12 (27.9)	0.25 (0.02–3.67)	
≥ 70	3 (7.0)	1.00	
Sex			n.s
Male	14 (32.6)	1.00	
Female	29 (67.4)	1.23 (0.34–4.49)	
Involved side			n.s
Right	21 (48.8)	1.67 (0.48–5.74)	
Left	22 (51.2)	1.00	
Body mass index, kg/m^2^			n.s
< 20.0	2 (4.7)	1.00	
20.0–24.9	15 (34.8)	1.34 (0.08–12.83)	
25.0–29.9	24 (55.8)	2.29 (0.17–30.96)	
≥ 30.0	2 (4.7)	3.75 (0.29–47.99)	
Lesion location			n.s
Medial femoral condyle	41 (65.1)	1.00	
Lateral femoral condyle	16 (25.4)	1.41 (0.25–7.86)	
Trochlea	6 (9.5)	2.20 (0.32–14.98)	
Lesion size, cm^2^			0.038
< 3.5	4 (9.3)	1.00	
3.5–5.5	18 (41.9)	2.67 (0.23–31.07)	
5.5–7.5	17 (39.5)	7.80 (0.65–93.81)	
≥ 7.5	4 (9.3)	13.4 (2.49–215.36)	
No. of SVF cells			0.021
< 7.0 × 10^7^	12 (27.9)	7.20 (0.64–81.54)	
7.0 × 10^7^–8.0 × 10^7^	25 (58.1)	1.80 (0.43–7.53)	
≥ 8.0 × 10^7^	6 (14.0)	1.00	

*SVF* Stromal vascular fraction, *CI* Confidence interval

Compared to patients with ≥ 8.0 × 10^7^ SVF cells, those with 7.0 × 10^7^ to 8.0 × 10^7^ SVF cells were 1.80 times more likely to have an unsatisfactory outcome (95% CI, 0.43–7.53). Meanwhile, patients with < 7.0 × 10^7^ SVF cells were 7.20 times more likely to have an unsatisfactory outcome (95% CI, 0.64–81.54). Age, sex, involved side, and BMI did not independently predict unsatisfactory outcomes after SVF implantation.

## Discussion

Although SVF-based treatment has demonstrated encouraging clinical efficacy for repairing articular cartilage in knee OA [5, 41, 42], we understand little about the preoperative factors that influence the treatment outcomes. This is the first study to assess the effects of various factors, including patient characteristics (age, sex, the involved side, and BMI), cartilage lesion size, and the number of SVF cells, on outcomes after SVF implantation. Understanding the factors associated with clinical outcomes will allow patients with OA to have more realistic expectations after undergoing SVF implantation for their knees.

Patient characteristics may serve as important selection criteria for cell-based repair strategies. For example, older age might significantly affect the SVF quality. Several studies have investigated this, with differing conclusions [2, 8, 12, 14, 43]. Yu et al. [43] found a positive correlation between the SVF yield and donor age (linear correlation coefficient *r* = 0.30). Furthermore, Buschmann et al. [8] evaluated the SVF yield from 30 donors, reporting that older patients (45–74 years) had a significantly lower SVF yield than middle-aged patients (38–44 years). In contrast, de Girolamo et al. [12] identified a significant positive correlation between age and cell yield, indicating that older donors had a larger cell harvest than younger donors. Conversely, Faustini et al. [14] performed a linear multiple regression analysis among 125 patients (mean age, 51.31 years; range, 15–87 years) to evaluate how donor age affects the SVF yield, reporting no influence. Finally, Alaaeddine et al. [2] compared the number of SVF cells among 58 adults (mean age, 39.4 years; range, 20–71 years) divided into four age groups (< 30, 30–39, 40–49, and ≥ 50) but found no differences among the groups (n.s.). They also found that the number of SVF cells did not differ by sex (n.s.). Similarly, our study found no correlation between the number of SVF cells and patient age or sex (Table 8), nor did we find differences in the mean VAS and MOCART scores among the age and sex subgroups (all n.s.; Tables 4 and 5). Although it remains unclear whether patient age or sex influences the number of SVF cells, we conclude that these variables do not influence the SVF implantation outcomes.

Obesity is a well-established risk factor for OA development and progression, especially in weight-bearing joints [13]. Furthermore, adipose-derived MSCs from overweight patients have a reduced proliferation rate, greater cell senescence, and reduced differentiation to multiple lineages, including chondrogenesis [32]. Some authors have reported a positive correlation between BMI and the SVF yield [2, 43], yet others have reported no correlation [4, 8, 14, 29]. We also found no correlation between the number of SVF cells and BMI (Table 9), nor did we find differences in the SVF implantation outcomes among the BMI groups (Table 6). Obesity [1] is defined as a BMI of ≥ 30.0 kg/m^2^; in this study, only three patients were classified into the obesity group, meaning that the number of SVF cells from these patients would not have influenced the outcomes. Therefore, further SVF implantation studies that compare outcomes among different BMI groups and include more patients with a BMI of ≥ 30.0 kg/m^2^ are needed to adequately evaluate the independent effect of BMI.

Strong correlations between the lesion size and outcomes of regenerative procedures for cartilage have been documented. For instance, Salzmann et al. [33] reported that microfracture surgeries are usually performed to treat lesions < 3 cm^2^ in size, and Knutsen et al. [24] indicated that full-thickness chondral defects < 4 cm^2^ respond better to microfracture surgery than lesions > 4 cm^2^. Furthermore, Koh et al. [25] evaluated 37 patients treated with MSC implantation, reporting that cartilage lesions > 5.4 cm^2^ had significantly worse clinical outcomes and less cartilage regeneration than those < 5.4 cm^2^. Kim et al. [21] also performed MSC implantation in 49 patients (55 knees) with knee OA and compared the outcomes based on the lesion size (< 3.0, 3.0–5.9, 6.0–8.9, and ≥ 9.0 cm^2^). They found significant differences in clinical outcomes among the groups and suggested that a 6.0 cm^2^ lesion was the upper size limit for obtaining encouraging outcomes after MSC implantation. Similar results were observed in the present study, where we assessed the patients based on the lesion size (< 3.5, 3.5–5.5, 5.5–7.5, and ≥ 7.5 cm^2^), finding a significant difference in mean MOCART scores among the groups (*P* = 0.007; Table 8) and a significant correlation between the MOCART score and the lesion size (Fig. 2C). Our study findings indicated that cartilage regeneration was less favourable after SVF implantation for larger cartilage lesions.

In addition, we found significant correlations between the mean VAS and MOCART scores 12 months after surgery (*P* = 0.002; Table 3), implying that until the cartilage regenerates, the pain levels are similar, but as regeneration occurs, the pain gradually improves. These results also suggest that at least 12 months is necessary for enough cartilage to regenerate to improve pain levels after SVF implantation. We also found a significant correlation between the 12-month VAS score and the lesion size (Fig. 2A). Together, these results suggest that postoperative pain decreases as the cartilage regenerates, and since the lesion size affects cartilage regeneration, the pain level is related to the lesion size. In addition, we found that the lesion size was an independent predictor of an unsatisfactory outcome after SVF implantation (*P* = 0.038; Table 12). Therefore, we conclude that the lesion size is a prognostic factor influencing SVF implantation outcomes.

One of the most important questions regarding regenerative treatment using SVF is the optimal number of SVF cells for favourable cartilage regeneration with satisfactory clinical outcomes. Several studies have reported promising results regarding intra-articular injections of SVF cells for knee OA treatment, and the average SVF doses in those studies varied from 1.4 × 10^7^ to 5.0 × 10^7^ cells [35]. Currently, whether the SVF amount affects the knee OA treatment outcome is debatable. For example, Fodor and Paulseth [15] stated that they did not observe a dose-dependent response to the SVF amount in their pilot study in eight patients with knee OA, where they performed an intra-articular injection of SVF (mean, 14.1 × 10^6^; range, 7.0 × 10^6^–14 × 10^6^). However, Simunec et al. [36] performed an intra-articular injection of SVF cells in 12 patients with knee OA, reporting a negative correlation between the number of administered cells and an improvement in the Knee injury and Osteoarthritis Outcome Score (KOOS) score (Pearson correlation coefficient: *r* =  − 0.27 at the 3-month follow-up and *r* =  − 0.35 at the 12-month follow-up), indicating that the lower the number of administered cells, the more the KOOS score improved. Meanwhile, other authors reported positive correlations between SVF-based treatment outcomes and the number of SVF cells. Tsubosaka et al. [38] compared the 12-month outcomes of 60 patients; 30 received an intra-articular injection with 2.5 × 10^7^ SVF cells (low-dose group), and 30 received an intra-articular injection of 5.0 × 10^7^ SVF cells (high-dose group). They reported that the 12-month postoperative pain and symptom subscale KOOS scores were significantly better in the high-dose group than in the low-dose group. However, they found no differences in the follow-up MRI evaluations between the two groups. Furthermore, Garza et al. [17] used freshly isolated SVF cells to treat knee OA, and the patients were allocated to a high-dose (3 × 10^7^ cells), low-dose (1.5 × 10^7^ cells), or placebo group. They found dose-dependent effects, with the higher dose producing more pronounced effects. Similar results were observed in our study. We used a mean of 7.4 × 10^7^ ± 4.8 × 10^6^ (range, 6.7 × 10^7^–8.5 × 10^7^) SVFs and identified a significant correlation between the MOCART score and the number of SVF cells (Fig. 2D). In addition, the mean MOCART scores differed among the four groups with differing SVF amounts (*P* = 0.001; Table 11). Notably, some variables (e.g., the degree of defect repair and defect filling, border zone integration, repair tissue surface, and repair tissue signal intensity) significantly differed among the four different SVF amount groups (*P* = 0.016, *P* = 0.018, *P* = 0.043, and *P* = 0.025, respectively; Table 11).

We attribute these results to the SVF characteristics. Unlike the cultured adipose-derived MSCs, which constitute a fairly homogenous cell population, adipose-derived SVF is a heterogeneous cell population containing regenerative cells, such as adipose-derived MSCs, macrophages, pericytes, fibroblasts, blood cells, vessel-forming cells (including endothelial and smooth muscle cells), and their progenitors [19]. Adipose-derived stem and stromal cells contribute to cartilage regeneration by tissue-specific differentiation, extracellular matrix secretion, and various immune-modulating factor secretions [7, 9, 45]. Fibroblasts secrete extracellular matrix components that positively influence cell adhesion, migration, and cell–matrix interactions [18]. Therefore, we speculated that we would identify significant differences between variables related to cartilage regeneration and the number of SVF cells, which we did (Table 11). We also speculated that macrophages in SVF, which secrete immunomodulatory factors and cytokines to induce anti-inflammatory effects, contribute to the significant difference between effusion and the number of SVF cells. This study found that the number of SVF cells was an independent predictor of unsatisfactory outcomes after SVF implantation (*P* = 0.021; Table 12). Therefore, we conclude that the number of SVF cells is a prognostic factor influencing the outcomes of SVF implantation.

This study has some limitations. First, the number of patients was relatively small, and the 12-month follow-up period was short. Thus, a larger series of cases with longer follow-up periods are required for a more accurate evaluation of the long-term outcomes and the prognostic factors associated with SVF implantation. However, given that no similar studies have been published, we believe these data are important. Second, although a follow-up MRI was performed to evaluate cartilage regeneration after SVF implantation, we did not conduct a histological evaluation to assess the quality of the regenerated cartilage. Second-look arthroscopy with a histological evaluation would help evaluate the quality of the repaired cartilage. Because SVF is a heterogeneous population of cells with variable growth potentials and distinct morphological and functional characteristics, the SVF quality required to achieve adequate cartilage regeneration should be identified to better predict SVF implantation outcomes. In this study, we found that the number of SVF cells was a prognostic factor influencing outcomes following SVF implantation. However, a future study should estimate other SVF characteristics that influence outcomes for a more accurate assessment of the influential prognostic factors. In addition, the optimal number of SVF cells should be determined by evaluating the effects of SVF cells on improved cartilage regeneration to achieve better clinical outcomes. Finally, a follow-up MRI was performed approximately 12 months postoperatively. However, the potential behaviour of regenerated cartilage over time remains unknown, and changes in the influential factors after 12 months cannot be predicted.

## Conclusion

The present study showed encouraging improvement in pain levels and cartilage regeneration after SVF implantation in patients with knee OA throughout the 12-month follow-up period; furthermore, the size of the cartilage lesion and the number of SVF cells significantly influenced patient outcomes following SVF implantation. These factors may serve as a more accurate screening tool, allowing surgeons to better assess which patients with knee OA are good candidates for SVF implantation.

## Abbreviations

OA: Osteoarthritis
MSCs: Mesenchymal stem cells
SVF: Stromal vascular fraction
MRI: Magnetic resonance imaging
BMI: Body mass index
VAS: Visual analogue scale
MOCART: Magnetic Resonance Observation of Cartilage Repair Tissue
CIs: Confidence intervals
KOOS: Knee injury and Osteoarthritis Outcome Score
SD: Standard deviation
